# Silicon Surface Tethered Polymer as Artificial Solid Electrolyte Interface

**DOI:** 10.1038/s41598-018-30000-z

**Published:** 2018-08-01

**Authors:** Brian H. Shen, Gabriel M. Veith, Wyatt E. Tenhaeff

**Affiliations:** 1 Department of Chemical Engineering, University of Rochester, Rochester, NY 14627 USA; 2 Materials Science and Technology Division, Oak Ridge National Laboratory, Oak Ridge, TN 37831 USA

## Abstract

We have developed a proof of concept electrode design to covalently graft poly(methyl methacrylate) brushes directly to silicon thin film electrodes *via* surface-initiated atom transfer radical polymerization. This polymer layer acts as a stable artificial solid electrolyte interface that enables surface passivation despite large volume changes during cycling. Thin polymer layers (75 nm) improve average first cycle coulombic efficiency from 62.4% in bare silicon electrodes to 76.3%. Average first cycle reversible capacity was improved from 3157 to 3935 mAh g^−1^, and average irreversible capacity was reduced from 2011 to 1020 mAh g^−1^. Electrochemical impedance spectroscopy performed on silicon electrodes showed that resistance from solid electrolyte interface formation increased from 79 to 1508 Ω in untreated silicon thin films over 26 cycles, while resistance growth was lower – from 98 to 498 Ω – in silicon films functionalized with PMMA brushes. The lower increase suggests enhanced surface passivation and lower electrolyte degradation. This work provides a pathway to develop artificial solid electrolyte interfaces synthesized under controlled reaction conditions.

## Introduction

Lithium-based energy storage technologies are attractive given their large cell voltages and power densities. While many materials have been explored for advanced lithium-based batteries, the graphite anode remains the commercially dominant choice mainly due to its ability to form a self-limiting solid electrolyte interface (SEI)^1^. This layer between the electrode and electrolyte is critical to long-term, stable performance of lithium ion batteries and prevents irreversible capacity loss by protecting the electrode from unfavorable side reactions and hindering continual electrolyte reduction, while simultaneously allowing lithium ion transport into the anode^2^. However, spontaneous formation of a SEI with long-term stability seems unique to graphite. The limitation of conventional graphite is its relatively low capacity compared to other materials. Silicon is a potential anode material to address low anode capacity. Lithiated silicon (Li_15_Si_4_) has a theoretical specific capacity of 3579 mAh∙g^−1^ versus 372 mAh∙g^−1^ for graphite at room temperature^3^. However, the volume of silicon expands and contracts by roughly 300% during (de)lithiation, resulting in suspected mechanical damage to the SEI^4^. As a result, accessible reversible capacity continually decreases as exposed bulk anode material electrochemically reduces electrolyte components during lithiation to reform the SEI, or as side reactions between electrolyte and surface oxide layers occur^5,6^.

One approach to addressing an unstable (non-passivating) SEI layer on silicon is the development of an artificial solid electrolyte interface^7^. This artificial SEI is ideally an ionically conductive and electrically insulating layer formed on the surface of the electrode and designed to passivate and protect the electrode in harsh cycling conditions, maintaining high capacity over many cycles. Once properly passivated, electrolyte near the electrode will be protected from further electrochemical degradation, and the electrode itself will be protected from etching and side reactions. Many approaches have been explored to improve passivation of silicon, which include embedding silicon particles in volume-confining conductive carbon matrices, using conductive organic additives, investigating alternative binders for traditional silicon composite electrodes, or coating silicon surfaces with passivating materials, such as LiPON, metal oxides or flourine^8–13^. While these techniques may be effective to varying degrees, silicon volume changes during cycling are still a challenge. Volume changes can lead to poor adhesion of silicon surfaces to binder or other matrices, and can damage and/or interfere with the uniformity of the coatings intended passivate the silicon^14,15^. Strong adhesion and coating stability is believed preferable, as exposure of silicon surfaces can lead to side reactions with electrolyte or poor ion transport to the active material^16–20^. Other approaches reported by Cui *et al*. accommodate silicon particle volume changes by allowing silicon expansion within an empty carbon hard shell of fixed volume^12,13^. However, these novel materials require several fabrication steps, including the use of a sacrificial SiO_2_ coating to template the particle morphology.

**Figure 1:**
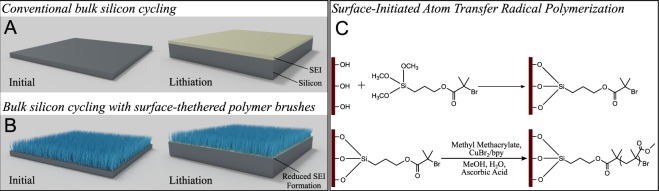
(**A**) Cartoon representation of bare bulk silicon (grey) thin films. The solid electrolyte interface (SEI, yellow) develops on the silicon surface during initial cycling. (**B**) Cartoon representation of cycling of bulk silicon functionalized with surface-tethered polymer brushes. Silicon surface passivation is enhanced, reducing the amount of SEI formed on the surface. (**C**) ATRP reaction scheme used to graft polymer chains to the silicon surface.

Our approach is to surface functionalize silicon anodes for lithium ion battery applications with surface-tethered polymer brushes *via* surface-initiated activators regenerated by electron transfer atom transfer radical polymerization (ARGET ATRP). ARGET ATRP is a “green” chemistry as it can be performed at room temperature in aqueous solvents and uses low ppm levels of catalyst and benign reducing agents, such as ascorbic acid^21^. In this technique, the reducing agent continuously regenerates the polymerization catalyst, which limits radical termination and relaxes strict oxygen free requirements for reaction conditions^22^. Because of its robustness, ARGET ATRP is a relatively simple technique enabling facile synthesis of polymer coatings on silicon electrodes. Poly(methyl methacrylate) (PMMA) brushes were synthesized, as PMMA is a commodity polymer often employed as a polymer gel electrolyte material for its good ionic conductivity and electrochemical stability^23,24^. In addition, polymerization of PMMA using (non)aqueous ATRP has been extensively documented^25,26^. The SEI layer synthesized by ATRP ARGET is a dense layer of PMMA brushes tethered at one end to the electrode surface. While polymeric artificial SEIs have been investigated, we know of few aqueous chemistries that result in a covalently bound polymer layer that can be grown from silicon surfaces at a predictable rate. In addition, the surface-tethered brushes can easily accommodate the large electrode volume fluctuations silicon undergoes during (de)lithiation and maintain silicon surface passivation. The result of this enhanced passivation is decreased additional SEI formation and surface side reactions during cycling, increasing the reversible capacity available toward silicon lithiation. As shown in Fig. 1A, thin film silicon generally forms a thick SEI layer (ca. 40 nm) upon lithiation from chemistries that result in irreversible capacity loss^27^. Addition of polymer, shown in Fig. 1B, helps mitigate irreversible capacity loss from SEI formation. The surface-initiated ATRP reaction scheme is presented in Fig. 1C. We have elected to surface functionalize 2D, flat thin film electrodes to enable better understanding of interfacial chemistries. Silicon thin films with thicknesses less than 50 nm are employed to limit strain-induced fracture during cycling^28,29^.

Half-cell cycling of PMMA coated silicon anodes shows an improvement of first cycle coulombic efficiency and average reversible capacity. Long term enhanced passivation of silicon is confirmed with potentiostatic electrochemical impedance spectroscopy. These findings will guide future development of surface-tethered polymeric artificial solid electrolyte interfaces synthesized *via* surface-initiated atom transfer radical polymerization.

## Results and Discussion

### Characterization

To determine growth rate of the PMMA brushes, ATRP synthesis on the silicon wafers were conducted for various reaction durations. After a given duration, the reactions were quenched by rinsing in solvent to remove residual reaction solution and soaked overnight to remove adsorbed organic material. The thickness of the brushes was characterized through step height measurements of the polymeric layer using profilometry. Data for the measured step heights are shown in Supplemental Fig. 1. From the data, the growth rate was 37.5 nm/hr. Profilometry scans were relatively flat and step-heights were easily reproducible, suggesting ATRP yielded coatings of uniform thicknesses. Experiments were also conducted to validate the surface initiation proposed in Fig. 1C. When the organosilane functionalization of Si was omitted (the first step in the scheme), no appreciable formation of PMMA brushes was observed on the Si surface. This suggests that polymerization occurs only on the initiator molecules attached to the silicon surface through the initial organosilane chemistry; thus, the polymer brushes are covalently tethered to the treated surfaces.

**Figure 2:**
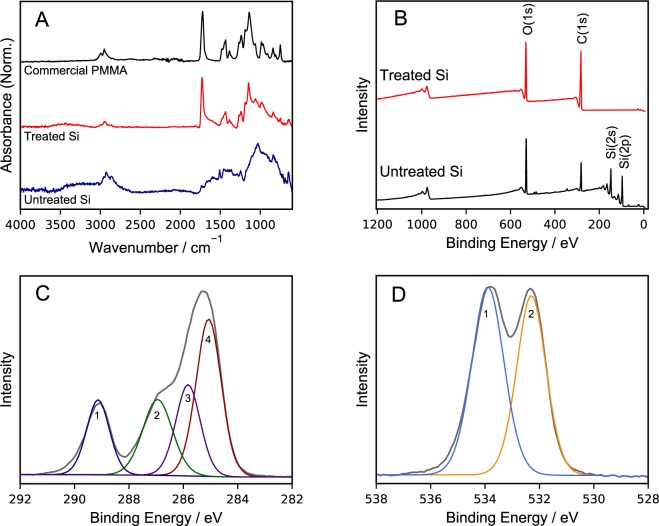
(**A**) FTIR-ATR spectra for (un)treated silicon to verify surface treatment. (**B**) XPS survey scans of (un)treated silicon. Also shown are high resolution XPS spectra (grey) and fits for (**C**) carbon and (**D**) oxygen.

To understand the surface chemistry of the silicon thin films, FTIR-ATR was performed. Figure 2A shows representative FTIR-ATR data of treated and untreated Si electrodes, as well as commercial PMMA for comparison. A vibrational mode at 1030 cm^−1^ for Si-O-Si stretching is observed in the spectrum for as-deposited Si electrodes^30^. The comparison of the Si-PMMA to commercial PMMA standard provides clear evidence of the successful synthesis of PMMA. In the Si-PMMA spectrum, the strong, sharp absorbance at 1728 cm^−1^ is attributed to the carbonyl (C=O) stretch characteristic of methacrylate functionalities^31^. Another main peak is observed at 1434 cm^−1^, which can be attributed to CH_2_ symmetric scissoring and O-CH_3_ deformation. Another prominent peak is observed at 1146 cm^−1^ for the C-O stretching in the OCH_3_ group^32^. As the PMMA-coated Si were soaked in acetone (a good solvent for PMMA^33^) overnight at room temperature, the measureable infrared absorbance of PMMA is strong support for the surface functionalization scheme presented in Fig. 1C, and further supports that the PMMA brushes are covalently bound to the Si surface.

Si electrodes were also analyzed *via* x-ray photoelectron spectroscopy to characterize the composition of the PMMA brushes. Survey and high-resolution spectra are presented in Fig. 2B–D. The elemental composition of the surface layer in untreated Si was determined to be 40.6% Si, 23.8% O, 35.6% C. The composition does not perfectly correlate to a native oxide on Si, which is expected given that these amorphous films are produced by high energy sputtering. Some of the carbon is likely adventitious carbon, but the presence of nonstoichiometric amorphous Si-O-C cannot be ruled out. In the high-resolution scan of the Si 2p region (shown in Supplemental Fig. S2), a Si^4+^ (SiO_2_) peak at 102.5 eV was observed that constitutes 19.8% of the total Si 2p region, suggesting an appreciable native oxide resides on the silicon surface. This concentration is comparable to reported findings on amorphous silicon thin films^34^.

The elemental composition of PMMA-coated silicon was determined to be 23.9% O and 76.1% C, yielding a C/O ratio of 3.2. The C/O ratio based for the theoretical PMMA composition is 2.5. Our PMMA films are somewhat enriched in carbon, but similar experimental C/O ratios for PMMA have been found in literature, ranging from 2.71 to 3.1, depending on the substrate’s exposure to adventitious carbon, treatments, or exposure to atmospheric conditions^35,36^. No peaks for bromine (68.7 eV) or copper (ca. 933 eV) are observed in the PMMA-coated silicon electrode survey scans. The lack of such peaks indicates the absence of impurities from the polymerization process and confirms the efficacy of the post-polymerization rinsing procedure. The complete attenuation of silicon peaks in the survey spectra after treatment (Fig. 2B) suggests the polymeric brushes completely occlude the silicon surface and are at least 10 nm thick, as expected from the profilometry measurements.

Deconvolution of the high resolution scans of the carbon and oxygen regions shown in Fig. 2C,D, respectively, reveal component peaks that are consistent with the expected chemical bonding of PMMA^37^. Tabulated data and group assignments for these high resolution scans are presented in Table 1. Slight discrepancies between experimental and theoretical group content are observed. However, the experimental content ratios closely match those found in literature^38–40^. Considering that the high-resolution spectra closely match literature, and silicon peak attenuation is observed in the survey scans, we can conclude that ATRP-ARGET is an effective approach to synthesizing compositionally well-defined PMMA brushes on amorphous Si electrode surfaces.

**Table 1:** XPS data for PMMA treated silicon thin films, along with comparison data from ^a^Briggs, *et. al*.^38^ and ^b^Kaczmerak, *et. al*.^39^.

Atom	Group Type	Exp. BE [eV]	Theo. BE [eV]^a^	Exp. Content [%]	Theo. Content [%]
C1s	O=**C**-O-CH_3_^b^	289.1	288.9	16.9	20.0
	-CH_2_-C(CH_3_)-COO**C**H_3_^b^	286.9	286.8	22.1	20.0
	**C**C(=O)R^a^	285.7	285.7	22.0	20.0
	**C**H_x_^a,b^, -**C**H_2_-CH_2_-**C**(CH_3_)-COOCH_3_^b^	285.0	285.0	39.0	40.0
O1s	-C=O^b^	533.9	533.6	53.4	50.0
	-**O**-CH_3_-C-**O**H^b^, -O-CH_3_^b^	532.3	532.1	46.6	50.0

### Electrochemical Cycling

**Figure 3:**
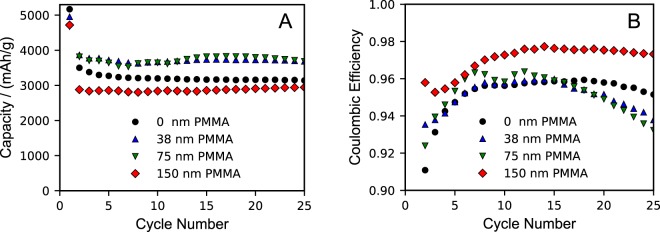
(**A**) Lithiation capacity and (**B**) coulombic efficiency (excluding first cycle) for silicon thin films with various coating thicknesses. Silicon electrodes were cycled against lithium metal using 1.0 M LiPF_6_ in 3:7 EC/DMC electrolyte at a (dis)charge rate of C/3 from 0.05 V to 1.5 V at room temperature.

After confirming that PMMA had been polymerized from the surface of thin film silicon and demonstrating the physical stability of PMMA brushes in solvents, the coated electrodes were integrated into coin cells. Cycling performance over 25 cycles at a C/3 charge & discharge current is presented in Fig. 3. Excluding the first cycle, the average lithiation capacity of untreated Si electrodes is 3178 ± 20.8 mAh g^−1^. Reversible capacity is observed to increase with PMMA layer thickness up to an optimum of 75 nm. Average lithiation capacity for silicon electrodes with PMMA brush lengths of 75 nm was 3730 ± 98.2 mAh g^−1^ over 25 cycles. Results for all brush lengths are summarized in Table 2.

**Table 2:** First and average cycling data as a function of coating thickness.

Coating Thickness	Avg. 1^st^ Lith. Cap.	Avg. 1^st^ Delith. Cap.	Cap. Under 0.3 V	1^st^ Coul. Eff.	Avg. Rev. Lith. Cap.	Avg. Coul. Eff.
nm	mAh g^−1^			%	mAh g^−1^	%
0	5168 ± 83	3228 ± 224	3157 ± 27	62.4 ± 2.8	3178 ± 21	95.8 ± 0.1
19	4989 ± 65	3393 ± 282	3379 ± 87	67.9 ± 4.9	3348 ± 19	96.7 ± 0.3
38	4955 ± 573	3514 ± 249	3935 ± 193	71.1 ± 3.2	3702 ± 37	95.6 ± 0.3
75	4672 ± 93	3564 ± 37	4024 ± 11	76.3 ± 2.3	3730 ± 98	95.7 ± 0.6
150	4654 ± 360	3121 ± 253	3100 ± 331	61.0 ± 12.0	2629 ± 26	97.9 ± 0.6
420	1333 ± 529	435 ± 152	709 ± 167	32.9 ± 1.7	3002 ± 175	96.4 ± 1.4
675	496 ± 18	54 ± 13	140 ± 90	10.9 ± 2.2	68 ± 5	84.2 ± 4.1

The PMMA brushes have a significant effect on the first-cycle electrochemical behavior of the Si films, particularly the delithiation capacity and coulombic efficiency. First cycle coulombic efficiency increases as the PMMA brush coating thickness increases from 0 to 75 nm. The untreated and PMMA-coated Si electrodes have comparable lithiation capacities, but the delithiation capacities increase with increasing thickness up to 75 nm, which explains the trend in first cycle coulombic efficiency. In bare electrodes, a first cycle coulombic efficiency of 64.4% is observed, which is consistent with previous studies of Si thin films^41,42^. With a 75 nm layer of PMMA, the average first cycle coulombic efficiency increased to 76.3%, which is comparable to other research utilizing thin film coatings on silicon based electrodes. Specifically, silicon thin films coated with amorphous carbon, polyethylene glycol, and fullerene reported initial coulombic efficiencies of 60%, 62%, and 80%^43–45^. Improvements in first cycling performance are important to consider since commercial lithium ion batteries are typically fabricated in a discharged state with cathodes limiting the cell capacity. Any lithium lost to SEI formation or side reactions is detrimental to overall capacity.

**Figure 4:**
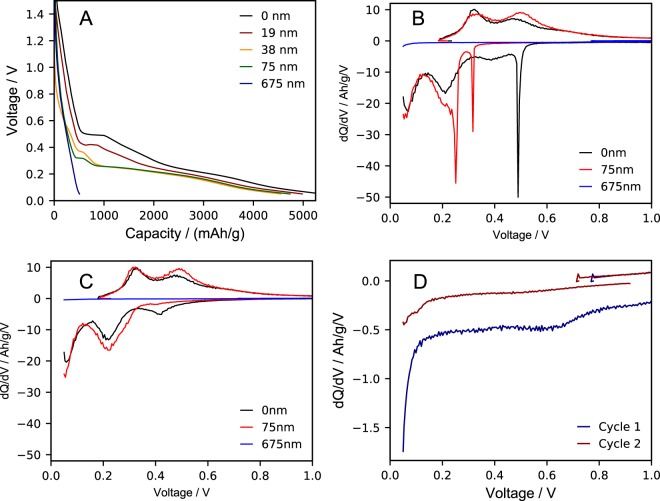
(**A**) First lithiation voltage profiles as a function of polymer coating thickness. Differential capacity curves during (**B**) the first cycle and (**C**) the second cycle of silicon coated with 0, 75 and 675 nm of PMMA, cycling at C/3. (**D**) Differential capacity curves during the first and second cycles of only the silicon electrodes coated with 675 nm of PMMA to highlight reduction peaks. All electrodes are cycled at of C/3 rate from 0.05 V to 1.5 V at room temperature.

Voltage profiles provide insight into why first cycle delithiation capacity and coulombic efficiency increases with polymer thickness up to 75 nm. Voltage profiles for lithiation at a C/3 rate are shown in Fig. 4A. Specific capacity obtained at voltages below 300 mV is observed to increase with PMMA coating layer thickness, up to 75 nm. Capacity at this potential range can be attributed to reversible lithiation of silicon, as amorphous silicon begins to transition to Li_*x*_Si (*x* = 0.0–2.0) at 250–300 mV^46^. The capacity above 300 mV is assumed to be irreversible due to electrolyte reduction, conversion of silicon oxide that may remain unpassivated by the PMMA brushes, reactions within the SEI layer, or other side reactions. The capacity below 300 mV is primarily due to lithiation of the silicon and should be reversible.

In untreated bare Si, the total lithiation capacity above 300 mV is 1985 mAh g^−1^. There is a significant electrolyte reduction plateau in the voltage profile and a corresponding peak in the differential capacity curve at 0.49 V. Below this plateau, additional irreversible capacity is observed before lithium insertion into silicon at 300 mV^47^. It is due to this substantial irreversible capacity that the first cycle coulombic efficiency is poor. The lithiation capacity below 300 mV is 3157 mAh g^−1^. Remarkably, the reversible capacity is very close to the measured first cycle delithiation capacity (3228 mAh g^−1^). As PMMA-coating thickness increases, irreversible capacity above 300 mV decreases while reversible lithiation capacity below 300 mV increases as reported in Table 2, suggesting less capacity is being lost to irreversible side reactions. This behavior is consistent with the increase in coulombic efficiency for PMMA-coating thicknesses up to 75 nm.

Improved silicon lithiation is also apparent from plots of differential capacity (dQ/dV). First and second cycle differential capacity curves are shown in Fig. 4B,C, respectively. In the first cycle, a sharp electrolyte reduction (SEI formation) peak for untreated films is observed at 0.49 V, while in films with a 75 nm PMMA layer, the magnitude of the peak is reduced and shifted to 0.31 V. The reduction peak is observed to be shifted lower in PMMA-treated electrodes as the thickness of the polymeric artificial SEI increases. We attribute this behavior to the PMMA layer occupying silicon surface active sites through the surface-tethered silane initiator and hindering electrolyte reduction at the electrode interface^48,49^. This behavior has been observed previously in silicon electrodes coated with LiPON thin films, where the magnitude of the electrolyte reduction peak decreased and potential shifted to lower values, from 0.47 V to 0.40 V as the LiPON thickness increased from 0 to 20 nm^50^. The electrolyte reduction peak at 0.31 V in PMMA-treated electrodes is also shallower than the peak observed in untreated silicon electrodes at 0.49 V, an indication that less capacity is lost to first cycle SEI formation, explaining the improved first cycle coulombic efficiency.

In addition to the electrolyte reduction peaks, a broad peak centered at ca. 0.4 V is observed during first cycle lithiation of the untreated silicon electrodes. This is significant capacity in the first lithiation step, but it appears to be largely reduced in the second step due to the irreversibility of the reaction. Functionalizing the Si electrode with PMMA brushes results in the disappearance of this feature, as observed for the differential capacity of the Si coated with 75 nm shown in Fig. 4B. These peaks are also largely absent in first cycle differential capacity curves for all silicon electrodes functionalized with PMMA brushes of various lengths, which are provided Supplemental Fig. S3. The first step of the functionalization of the Si electrodes is the reaction of the organosilane with reactive surface silanol (Si-OH) groups, which would otherwise react irreversibly in the first lithiation step^6,34^. This reaction results in surface siloxanes (Si-O-Si), which have been reported to reduce irreversible capacities^51,52^. Thus, we attribute the feature at 0.40 V to reaction between lithium and SiO_2_ or surface silanols. For the most part, this feature is eliminated in functionalized electrodes, as surface silanols are occupied by the silane initiator shown in the ATRP ARGET reaction. This suggests that less irreversible capacity loss is observed to these surface side reactions after functionalization, which helps to improve first cycle coulombic efficiency.

Lithiation peaks for both untreated and 75 nm PMMA coated anodes are observed at 0.22 V and 0.06 V for the first two cycles. The first lithiation process from 0.30–0.25 V can be attributed to the transition of the amorphous silicon to a phase consisting of Li_2.0_Si, Si-Si clusters and Si networks^53,54^. First lithiation for electrodes coated with PMMA around 0.22 V occurs around two distinct electrochemical potentials, matching previous literature showing this first lithiation is possibly two distinct processes, with a broad peak at 0.22 V and a sharp peak at 0.25 V^55^. This can be attributed to multiple phases or non-uniform lithiation events^56^. Delithiation occurs at similar potentials for both the untreated and PMMA-coated electrodes, which suggests the PMMA brush layers up to 75 nm in thickness does not introduce significant kinetic limitations to lithiation^57^. In fact, the integrated areas of the lithiation peaks are actually larger for the 75 nm PMMA-coated Si electrodes, showing that there is more available reversible capacity. This behavior continues into the second cycle, where again the lithiation peaks at 0.22 V and 0.06 V are larger in electrodes with a 75 nm coating of PMMA than in those without, which is an indication that more capacity is dedicated to lithiation of the silicon, rather than in irreversible side reactions. Additional cycling of silicon shows the current observed in silicon lithiation at 0.06 and 0.26 V increases with polymer coating thickness up to 75 nm (Supplemental Fig. S3).

It is important to consider data in Table 2, which reveal that while the first cycle coulombic efficiency in silicon electrodes improves as the PMMA brush coating thickness increases to 75 nm, it decreases beyond thicknesses of 75 nm. For example, while the electrodes with 150 nm PMMA appears to have good cycling performance, the first cycle coulombic efficiency and capacity retention are poor in comparison to electrodes with 75 nm. This observation is especially evident in films with very thick coatings. For example, first cycle coulombic efficiency is only 10.9% in films with a polymer brush film 675 nm thick. Differential capacity curves of electrodes with thicker PMMA brush coatings elucidate the reason for this behavior. Relative to the untreated Si, the reduction peaks in the differential capacity curves are shallower and overpotentials are much higher, which explains the poor coulombic efficiency for these samples. Expanded differential capacity curves for silicon electrodes with a coating of 675 nm thick are shown in Fig. 4D. In the first cycle, a broad, shallow feature around 0.6 V is observed, and does not appear again in the second cycle. This feature is likely due to electrolyte reduction nearest the electrode surface, suggesting that the thick PMMA layer is excluding the electrolyte from the Si interface. This results in less irreversible capacity from electrolyte reduction, but the trade-off is higher resistance as less electrolyte is available within the brush network to transport the ions to/from the interface as the layer thickness increases beyond 75 nm. This resistance increases the overpotential, and the cell reaches its lower voltage limit (50 mV) before full lithiation of the electrode can occur. The magnitude of this overpotential can also be observed in the relaxation of the cell after lithiation. The cycling protocol allows a 5-minute voltage relaxation between that lithiation and delithiation. For Si coated with 675 nm brushes, the cell open circuit potential relaxes to 0.82 V, which is further indication that limited lithiation occurred. Thus, electrodes with PMMA coatings thicker than 75 nm may exhibit better longevity, but will cycle with lower average reversible capacity. In high energy density cells, near 100% utilization of the Si anode is an important metric in order to minimize the mass and volume of the electrode.

To understand the contribution of the covalently tethered silane initiator to the electrochemical behavior, Si that were reacted with just the organosilane were also cycled in half cells. First cycle voltage profiles of these cells are compared to bare, untreated Si electrodes in Supplemental Fig. S3C. More capacity is observed above 0.3 V for the silane-treated electrodes, presumably due to irreversible reactions with the organosilane layer. The silane-treated electrodes have a slightly higher initial lithiation capacity than the untreated electrodes (approximately 101 mAh g^−1^), which may be attributed to these irreversible reactions. They also exhibit a lower initial delithiation capacity. We posit that much of the capacity loss in the silane-treated electrodes is partially due to the halogen species in the silane. For the purposes of the ATRP reaction, this species is intended to be readily reduced to allow chain propagation. Thus, the bromine-termination on the silane may actually serve as a site to promote electrolyte reduction, resulting in additional irreversible capacity loss. Thus, the improvements in first cycle performance discussed earlier can be largely attributed to the influence of the PMMA brush layer, rather than the silane initiator itself.

### Impedance

**Figure 5:**
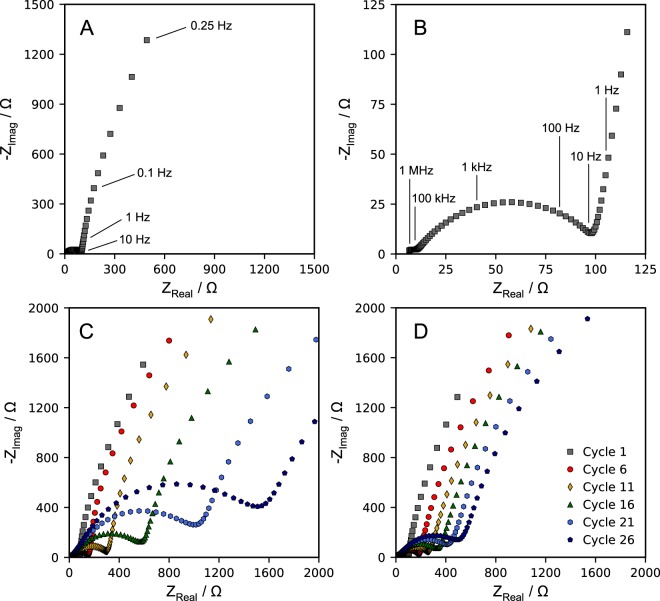
(**A**) EIS spectra of silicon electrode with 75 nm thick PMMA brush layer, acquired after first lithiation to 50 mV. (**B**) Higher frequency assignments of the impedance data shown in panel A. Three electrode PEIS measurements were taken during cycling of (**C**) bare silicon anodes and (**D**) silicon anodes coated with a 75 nm thick PMMA brush layer. The silicon anodes acted as the working electrode, lithium foil acted as counter and reference electrode.

To investigate the development of the SEI over time and long-term effects of PMMA on silicon electrodes, electrochemical impedance spectroscopy (EIS) was performed over an extended number of cycles at 25 °C. Three electrode cells were employed using silicon thin films as the working electrode (WE) and lithium metal as the counter and reference electrodes (CE & RE). The silicon electrodes were cycled against lithium metal at C/3 from 0.05 V to 1.5 V. Every five cycles, the silicon electrodes were lithiated to and potentiostatically held at 0.05 V before performing impedance spectroscopy with an amplitude of 10 mV. Impedance data as a function of cycle number are shown in Fig. 5. The impedance results can be described by the following equivalent circuit, where the bracketed elements are in parallel: R_electrolyt*e*_ - [R - CPE]_high_ - [R - CPE]_medium_ - [R - CPE]_low_. The most prominent features in the spectra are (1) a large, medium-frequency, potential-dependent semicircle (100 kHz to 10 Hz) and (2) a low-frequency region (<10 Hz).

The medium-frequency semicircles span a frequency range of 100 kHz to 10 Hz. The semicircle, emphasized in Fig. 5B, is somewhat depressed and appears to arise from a combination of two processes: charge transfer and lithium transport in the surface film on the silicon^58–60^. This surface film can be the PMMA brush layer or the SEI formed during first cycle lithiation. Before first lithiation, the impedance spectrum lacks a distinct semicircle at these frequencies. This semicircle, however, becomes more prominent after cycling, supporting that this impedance feature arises from the surface film. The low frequency (<10 Hz) feature emphasized in Fig. 5A can be attributed to the semi-infinite diffusion of lithium ions in the bulk silicon thin film^61,62^.

For untreated electrodes, the medium-frequency semicircle (surface layer) appears to grow considerably over the course of many cycles, as shown in Fig. 5C. After the first lithiation to 0.05 V, the resistance is observed to be 79 Ω. After 25 cycles, the resistance increased to 1508 Ω. As more solid electrolyte interface components form on the surface of the electrode, the resistance increases substantially. In contrast, treated silicon electrodes exhibit reduced resistance growth (Fig. 5D). Resistance after first lithiation is measured at 98 Ω. After 25 cycles, the resistance is measured at 498 Ω. The increase in resistance over the same number of cycles is much less pronounced compared to that of untreated electrodes. This suggests that the addition of a thin artificial SEI enhances silicon surface passivation and that passivation layers are relatively stable during cycling. Overall, impedance data suggest that surface tethered PMMA brushes have a beneficial long-term effect on silicon electrode performance.

Additional impedance data collected before and after lithiation of bare and PMMA-coated electrodes are provided in Supplemental Figs S4 and S5, which show development of the medium frequency semicircle after first lithiation. In addition, the medium frequency semicircle appears potential dependent. As the potential is lowered during the lithiation process, the total resistance associated with the semicircle decreases. It is difficult to quantitatively model the semicircles in the spectra due to deviations from ideal behavior and significant overlap of the frequencies associated with these processes. Impedance data of treated silicon films as a function of potential are shown in Supplemental Fig. S6.

We acknowledge there is a small, partial semi-circle present with a characteristic frequency of 1 MHz in the impedance spectra shown in Fig. 5B and Supplemental Figs S4 and S5. This feature is potential independent and constant throughout all cycling experiments. We attribute this feature to distortions caused by electrochemical (separator diameter relative to electrode diameter) and geometric (working and counter electrode alignment) asymmetry that occurred during three-electrode cell assembly, as well as the reference electrode geometry and positioning relative to the working and counter electrodes^63,64^.

### Post-Cycling FTIR

**Figure 6:**
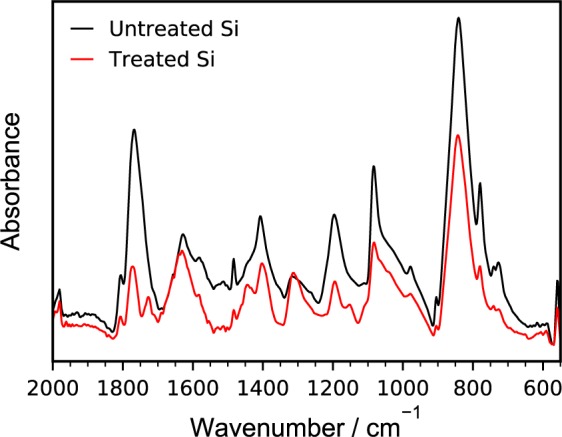
FTIR-ATR spectra of untreated and treated (75 nm PMMA) silicon electrodes that were lithiated to 0.05 V and then delithiated to 1.5 V at C/3 using 1.2 M LiPF_6_ in 3:7 EC/DMC electrolyte. Electrodes were rinsed with dimethyl carbonate and dried at 80 °C.

To further understand the effect of the PMMA coatings on silicon SEI formation and composition, FTIR-ATR characterizations were performed on bare and PMMA-coated (75 nm) silicon electrodes after cycling. Electrodes were lithiated to 0.05 V at C/3 in 1.2 M LiPF_6_ in 3:7 EC:DMC and were then delithiated to 1.5 V to establish an SEI. The electrodes were rinsed in DMC and then dried at 80 °C overnight. Figure 6 show the comparison of post-mortem FTIR-ATR spectra from PMMA-coated and untreated silicon electrodes. Both spectra show peaks typically associated with electrolyte reduction products. The most prominent of the peaks is observed at 842 cm^−1^, which is attributed to the P-F vibrations in LiPF_6_ decomposition products^65^. Peaks associated with ethylene carbonate decomposition products are observed at 1805 cm^−1^ (C=O st.), 1770 cm^−1^ (C=O st.), and 1200 cm^−1^ (C-O asym. st.)^66^. Peaks associated with ethylene carbonate reduction products are observed as well. Peaks at 1630 cm^−1^ (C=O asym st.), 1405 cm^−1^ (CH_2_ bend), 1315 cm^−1^ (C=O sym. st.), and 1083 cm^−1^ (C-O st.) can be attributed to (CH_2_OCO_2_Li)_2_, a common reduction product of ethylene carbonate based electrolytes, and the peak at 1483 cm^−1^ can be attributed to Li_2_CO_3_^67^.

The FTIR-ATR spectrum of the PMMA-coated silicon electrode after cycling reveals additional peaks that are typically attributed to bulk PMMA and previously appear in Fig. 2, suggesting that at least some fraction of the PMMA coating remains intact during the cycling. The polymer acrylate group vibration can be observed at 1729 cm^−1^. CH_2_ symmetric (scissor) and O-CH_3_ deformation in PMMA is observed at 1448 cm^−1^. Finally, PMMA C-O stretching is observed at 1150 cm^−1^. The presence of these peaks after cycling suggests good stability of the polymer layer during (de)lithiation of the electrode and first cycle SEI formation. The percentage of PMMA that reacts with SEI components has not been quantified.

Comparing the FTIR spectra shows that the PMMA coating has a marked effect on first cycle SEI formation on silicon. Under the same baseline, the intensity of the cycled PMMA-coated electrode spectrum is lower than that of the cycled bare electrode spectrum. The spectra suggest that there is a reduced presence of P-F and ethylene carbonate decomposition/reduction products on the surface of the PMMA-coated electrodes relative to that of the untreated electrodes. The largest decreases are in the peaks associated with ethylene decomposition products (1770 and 1805 cm^−1^) and P-F (842 cm^−1^). These decreases are an indication that while the PMMA does not act to completely eliminate additional SEI formation during the first cycle, it does serve to limit degradation of the electrolyte. Thus, in terms of first cycle performance, the PMMA reduces irreversible capacity loss, which is in agreement with earlier cycling data. This observation is also in agreement with the impedance spectroscopy data, which also suggest persistence of the PMMA and a reduction in the formation/adsorption of organic species on the surfaces of the electrodes over many cycles.

## Conclusions

In this work, we have presented facile, functionalization of silicon based electrodes with PMMA brushes as artificial SEI’s *via* ATRP. Surface tethered PMMA brushes were shown to mitigate first cycle capacity loss and improve first cycle coulombic efficiency from 62.4% with no treatment to 76.3% with 75 nm of PMMA on the silicon surface. Average reversible lithiation capacity increased from 3157 ± 27 mAh g^−1^ in silicon electrodes with no treatment to 4024 ± 11 mAh g^−1^ in silicon electrodes with a 75 nm PMMA film. Longer term effects were also observed through EIS, which demonstrated that the PMMA brushes reduce SEI development during cycling. After 25 cycles, resistance observed in untreated electrodes increased from 79 to 1508 Ω. In similar conditions, resistance observed in silicon anodes coated with 75 nm of PMMA only increased from 98 to 498 Ω. Post-cycling FTIR shows the PMMA is electrochemically stable during cycling and helps to reduce decomposition of ethylene carbonate and salt decomposition. Future work will investigate ATRP synthesis of artificial SEI composed of ionically conductive polymers to address lithium transport limitations from thicker brush layers.

## Experimental

### Synthesis Methods

Amorphous silicon was deposited onto copper foil (99.8% purity) to a thickness of 50 nm *via* radio-frequency (RF) magnetron sputtering of a two-inch Si target (undoped, 99.999% purity, Kurt J. Lesker) in a custom-built deposition chamber. Deposition was performed with a forward power of 90 W, 7.5 sccm flow of Ar and chamber pressure of 7.5 mTorr at a target-substrate distance of 7 cm. The silicon thin films were then reacted with an organosilane bearing the ATRP initiator. They were submerged in a solution of 10 µL of (3-trimethoxysilyl)propyl 2-bromo-2-methylpropionate (Gelest) and 24 mL of toluene and allowed to react for 16 hours at 65 °C. The organosilane-reacted Si film was then rinsed with methanol and introduced to a vial containing the polymerization solution consisting of deionized water (2.40 g), methyl methacrylate (99%, ≤30 ppm MEHQ inhibitor, 11.3 g), methanol (99.8%, 7.60 g), copper (II) bromide (99%, 4.40 mg), 2,2′-bipyridine (≥ 99%, 30.9 mg), and L-ascorbic acid (Reagent Grade, 34.9 mg). Methyl methacrylate was thrice passed through a DH-4 deinhibitor column prior to use. The polymerization solution was stirred at 500 rpm at room temperature. All chemicals were purchased from Sigma Aldrich and used as-received unless otherwise noted.

### Film Characterization

Prior to film characterization, samples were soaked in acetone overnight, thrice rinsed with acetone, and dried at 100 °C to ensure the polymer brushes were covalently attached to the silicon surface rather than simply adsorbed onto the surface.

FTIR was performed using a Bruker Alpha spectrometer using an attenuated total reflectance unit with a monolithic diamond crystal and 45° incident angle (Bruker Platinum ATR). Measurements were collected from 400 to 4000 cm^−1^ with a 4 cm^−1^ resolution using a RT-DLATGS detector with a KBr beam splitter. Spectra of the copper foil substrates served as backgrounds. A total of 64 scans were integrated to improve the signal-to-noise ratio. The spectra were baseline corrected and normalized for comparison purposes.

XPS was performed on a Kratos Axis Ultra spectrometer with a monochromatized Al Ka source operating at 15 kV and 10 mA. Samples were attached to the sample rod *via* double-sided carbon tape. A total of three scans from 1200 to −5 eV with a 1 eV step were integrated for survey spectra. The survey scan pass energy was chosen at 160 eV. Binding energies were calibrated based on the C 1 s peak at 284.8 eV. A total of ten scans were integrated for high resolution spectra for C 1 s, O 1 s, and Si 2p with a pass energy of 20 eV. Peaks were deconvoluted in CasaXPS software. A Gaussian-Lorentzian mix was used to determine peak shape and Shirley method was used to model the baseline.

To determine the rate of polymer growth, PMMA was grown from silicon wafer surfaces and underwent the aforementioned post-polymerization treatment. The PMMA film was scratched with a stainless-steel blade to form a clean step. Step height was determined using a profilometer (Model 3030, Dektak).

### Electrochemical Characterization

Coin cells were assembled inside an Ar-filled glovebox with copper-supported Si thin films as the working electrode and lithium metal as the counter/reference electrode. The silicon electrodes and separators were dried at 80 °C before battery assembly. A high-strength, nonwoven, aromatic polyamide (aramid) fiber based separator (Gold 40, Dreamweaver) was used to separate the two electrodes. The composition of the liquid electrolyte was 1.2 M LiPF_6_ in 3:7 (v:v) EC:DMC (Novolyte, BASF), and 100 μL of the electrolyte was added to the separator. The coin cells were cycled galvanostatically from 0.05 to 1.5 V at a C/3 rate on an Maccor Series 4000 cycler. Temperature was maintained at 25 °C.

To isolate the response of the working electrode in electrochemical impedance spectroscopy, three-electrode split cells were employed (EQ-3ESTC15, MTI Corporation). The configuration in these cells consisted of Si as the working electrode (14.3 mm dia.), lithium metal foil as the counter electrode (14.3 mm dia.), and a small piece of lithium foil (ca. 2 mm × 2 mm) attached to the end of a thin strip of copper foil served as the reference electrode. The reference lithium was positioned off-center between the working and counter electrodes. Two separators (19 mm dia., Gold 40, Dreamweaver) were used to prevent contact among the three electrodes. Cells were cycled from 0.05 V to 1.0 V at C/3 (SP-200, BioLogic Instruments). For each impedance measurement, the silicon (WE) was lithiated to 0.05 V and potentiostatically held there for two hours before performing EIS, with a frequency sweep from 7 MHz to 25 mHz and an amplitude of 10 mV.

## Electronic supplementary material

Supplementary Information
