# Supplementary material for: Silicon Surface Tethered Polymer as Artificial Solid Electrolyte Interface
Source: Sci Rep. 2018 Aug 1;8:11549. doi: 10.1038/s41598-018-30000-z (PMC6070503; doi:10.1038/s41598-018-30000-z)

Supplementary Information: Silicon Surface Tethered Polymer as Artificial Solid Electrolyte Interface

Brian H. Shen[a], *Gabriel M. Veith*[b], *Wyatt E. Tenhaeff*[a,†]

[a] Department of Chemical Engineering, University of Rochester, Rochester, NY 14627, USA.

[b] Materials Science and Technology Division, Oak Ridge National Laboratory, Oak Ridge, TN 37831, USA.

† Corresponding Author
Email: wyatt.tenhaeff@rochester.edu

Figure S1. Thickness of PMMA brushes synthesized by ATRP on Si wafers as a function of reaction time.

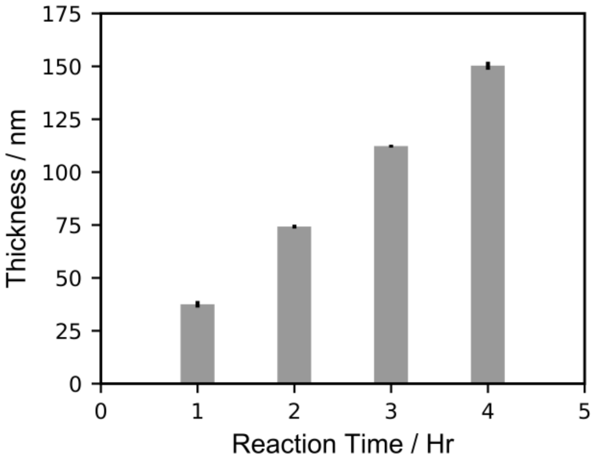

Figure S2. High resolution XPS spectrum of the as-deposited, bare silicon electrode. Silicon thin films (50 nm) were prepared by RF sputtering in Ar(g) onto a copper foil substrate.

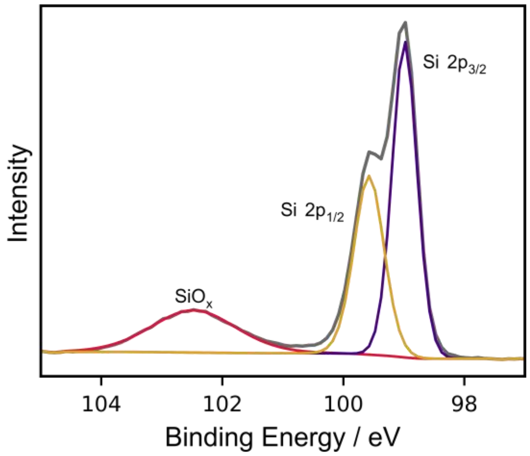

Figure S3. A) First cycle differential capacity curves for silicon electrodes with varying PMMA brush coating thicknesses. B) First cycle differential capacity curves for silicon electrodes with PMMA brush coating thicknesses of 420 nm and 675 nm. Shown to emphasize peaks. C) First cycle voltage profiles of untreated and silane-treated silicon electrodes cycled at C/3.

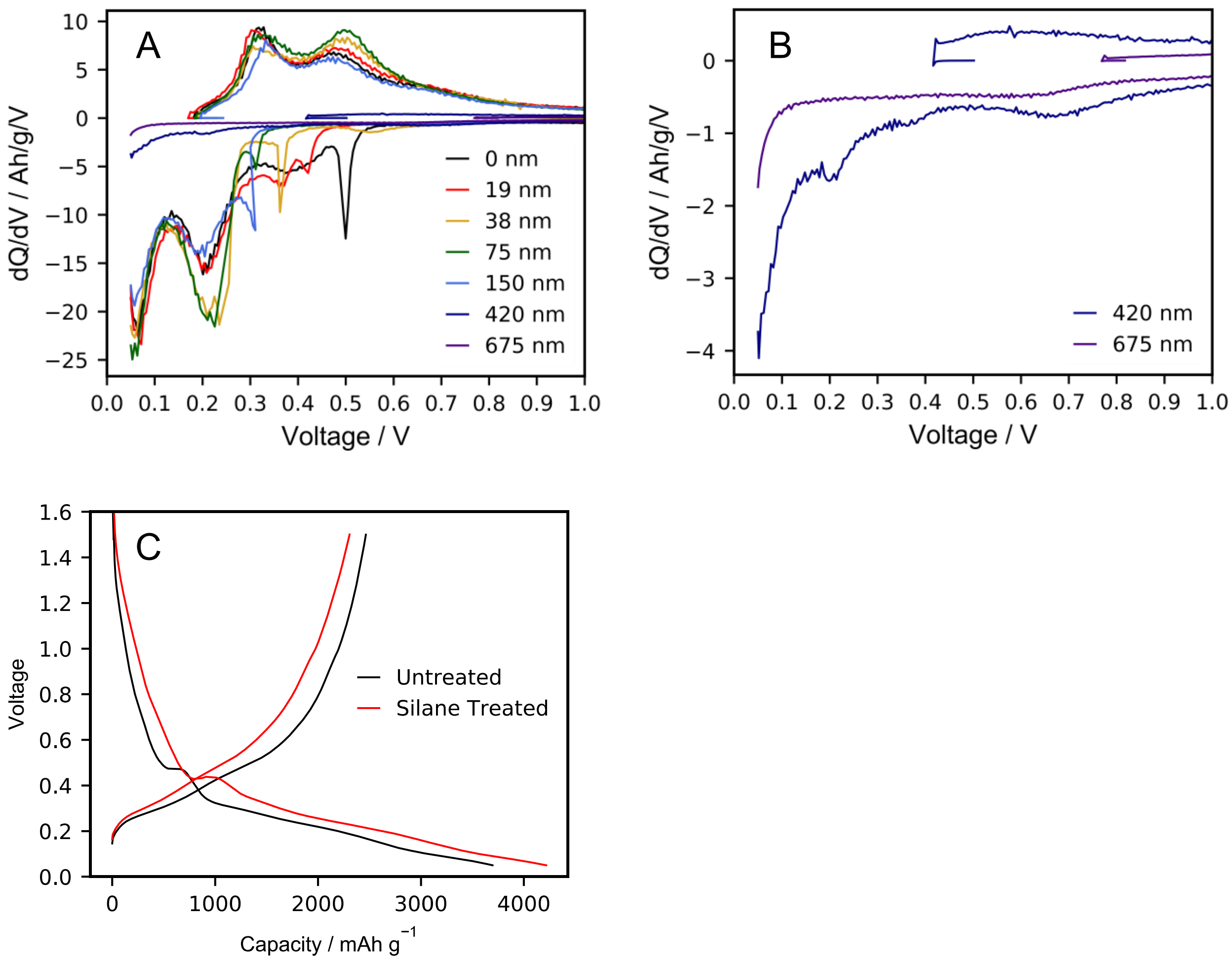

Figure S4. (A-B) Impedance spectra for as-deposited, bare silicon electrodes before lithiation at OCV, showing (A) all frequencies and (B) high-medium frequencies. (C-D) Impedance spectra for as-deposited, bare silicon electrodes after first lithiation at a potential of 50 mV, showing (C) all frequencies and (D) high-medium frequencies.

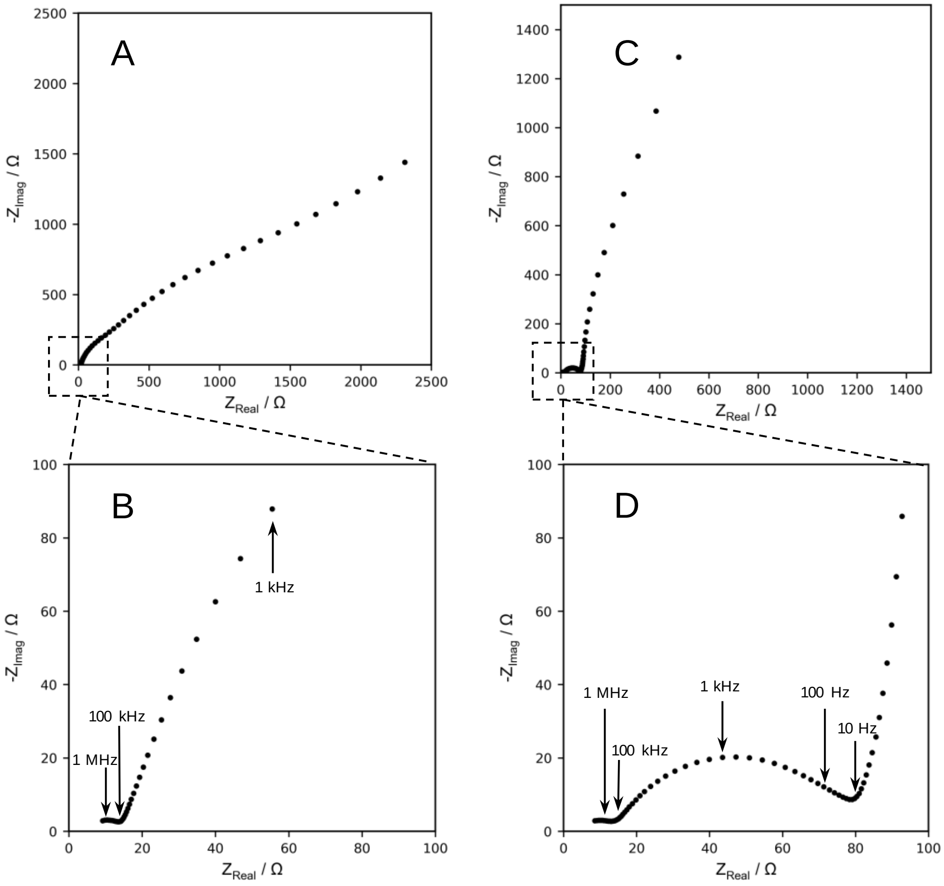

Figure S5. (A-B) Impedance spectra for silicon electrodes coated with 75 nm PMMA brushes before lithiation showing (A) all frequencies and (B) high-medium frequencies. (C-D) Impedance spectra for silicon electrodes coated with 75 nm PMMA brushes after first lithiation at a potential of 50 mV, showing (C) all frequencies and (D) high-medium frequencies.

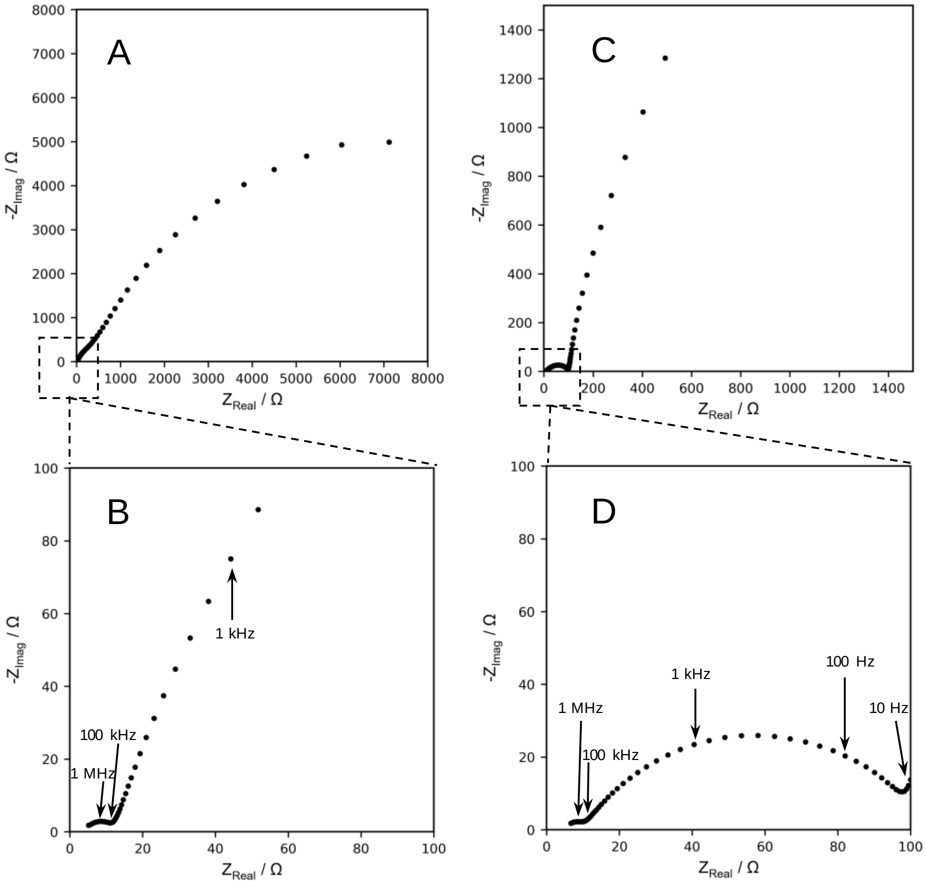

Figure S6. A) Nyquist plots and B) Bode plots for silicon electrodes coated with 150 nm PMMA brushes as a function of delithiation potential. Three electrode cells were assembled. The silicon electrode was the WE, lithium foil was the CE, and a small piece of lithium was the RE. The silicon was lithiated to 0.050 V and held at that potential for two hours prior to impedance measurements. This procedure was repeated as the cell potential was raised incrementally and the silicon was delithiated.

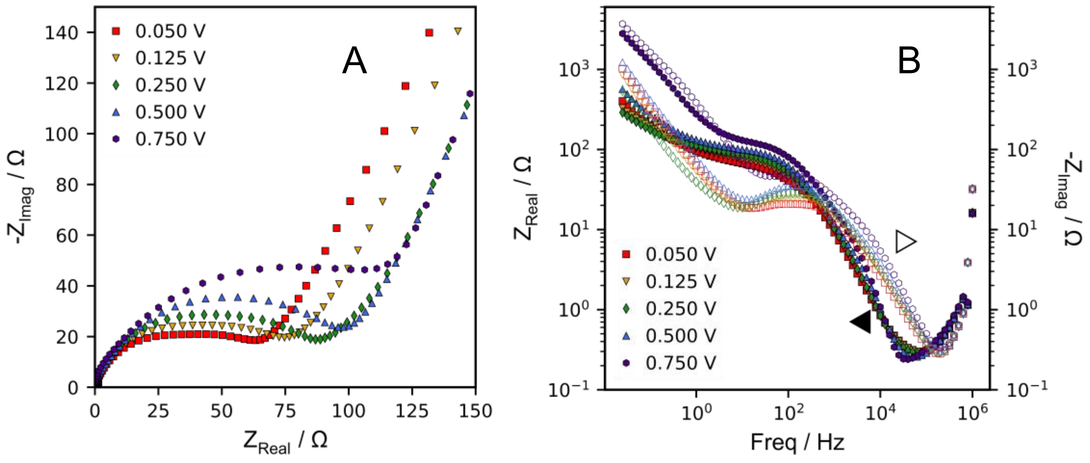

Supplement: Supplementary file 1 — Supplementary Information [file 41598_2018_30000_MOESM1_ESM.pdf]
